# Detrusorrhaphy during Robot-Assisted Radical Prostatectomy: Early Recovery of Urinary Continence and Surgical Technique

**DOI:** 10.1155/2019/1528142

**Published:** 2019-02-12

**Authors:** Tae Young Shin, Yong Seong Lee

**Affiliations:** Department of Urology, Hallym University Sacred Heart Hospital, Hallym University, College of Medicine, Anyang, Republic of Korea

## Abstract

Robot-assisted radical prostatectomy (RARP) has largely replaced open radical prostatectomy as the standard surgical treatment for prostate cancer. However, postoperative urinary incontinence still persists and has a significant impact on quality of life. We report the superior results of the detrusorrhaphy technique during RARP that helps achieve early continence. Our prospective study involved 95 consecutive patients who underwent RARP between March 2015 and May 2017; fifty patients underwent RARP using the new detrusorrhaphy technique (group 1) and 45 underwent standard RARP (group 2). The postoperative oncological and functional outcomes were compared between the two groups. The postoperative continence was assessed at 0 day, 1 week, 4 weeks, 8–12 weeks, and 6 months after catheter removal. Continence was defined as the use of no pad over a 24 h period. Mean operative time in groups 1 and 2 were 250 and 220 min, respectively. Intraoperative complications were not encountered in any patient. The continence rates after catheter removal in groups 1 and 2 were 68% and 0% at 0 day, 78% and 17.8% at 1 week, 86% and 64.4% at 4 weeks, 92% and 73.3% at 8–12 weeks, and 100% and 91.1% at 6 months, respectively. In the multivariate analysis, the nerve sparing technique, D'Amico risk groups, and prostate volume were involved in the early recovery of urinary continence. The detrusorrhaphy technique is simple, safe, and feasible, which helped achieve earlier continence. It showed significantly better outcomes than those achieved with the standard RARP technique in terms of urinary incontinence. Nevertheless, our findings need to be validated in further studies.

## 1. Introduction

Over the last decade, robot-assisted radical prostatectomy (RARP) has been increasingly adopted as a surgical treatment option for patients with localized prostate cancer [1, 2]. RARP achieves excellent oncological outcomes and is associated with a low risk of complications [3, 4]. Posttreatment quality of life (QoL) of patients with prostate cancer with respect to the recovery of urinary and sexual function has been an important area of study over the last decade. In particular, incontinence, temporary or permanent, is the most troublesome adverse complication of prostatectomy [5]. The incidence of incontinence at 12 months after surgery is estimated to range from 69% to 96% and can markedly impair the QoL of patients, particularly of those who are younger and more active [6]. However, this long recovery period is troublesome and psychologically distressful for patients. Moreover, it also has financial implications because of the need for medications and additional surgical procedures, such as the placement of a sling, urethral bulking agents, or an artificial urinary sphincter [7].

Many surgical adaptations that hasten continence recovery have been published. Various techniques have been used as long as oncological outcomes are not compromised [8–11].

We identified and implemented key operative techniques during RARP that are essential to achieve early continence in a step-wise manner. First, we preserved the periprostatic structures, such as the endopelvic fascia, deep dorsal vein, and puboprostatic ligament. Second, we performed dissection of the vas deferens, seminal vesicles, and pedicles using athermal means and with no or minimal clipping. Third, we secured maximum length of the urethra and performed the detrusorrhaphy technique, which is a zigzag flap of detrusor muscles. Herein, we present the detrusorrhaphy technique to achieve earlier recovery of urinary continence and assess the postoperative outcomes.

## 2. Materials and Methods

### 2.1. Study Design

This is a prospective study involving 95 consecutive patients who underwent RARP between March 2015 and May 2017; fifty patients underwent RARP using the new detrusorrhaphy technique (group 1) and 45 underwent standard RARP (group 2). Our prospective study was planned by dividing the groups with and without the detrusorrhaphy technique (50:50) among 100 consecutive patients, and the order numbers are summarized based on the previously prepared random number table. The random number table was applied sequentially to the patients who permitted the consent form. In group 2, there were 5 missing values. Due to their personal issues, two patients withdrew their consents after the operation, and three of the patients from the other country could not follow up and manage after our surgical treatment. The enrolled patients were divided into two subgroups in group 1 (enrolled numbers 1–25, 26–50) and group 2 (enrolled numbers 1–23, 24–45) according to a time criterion to compare the learning curve. The data were collected in a customized database and analyzed. The study protocol was approved by the University Hospital Ethics Committee. Indications for RARP are identical to those for open prostatectomy. RARP can be performed in patients with prostate cancer who have clinical stage ≤ T3b (seminal vesicles invasion) disease with no clinical or radiographic evidence of metastasis. Exclusion criteria were patients who received prior radiation therapy and those with a previous history of urethral stricture and urinary incontinence. One surgeon (YS LEE) with an experience of > 500 RARPs performed these surgeries.

### 2.2. Surgical Technique

Ninety-three patients underwent transperitoneal RARP, and two patients who had a prior history of abdominal operation underwent extraperitoneal RARP. Patient position and port placement were standard and are previously described [11]. We described the main surgical steps of RARP.

#### 2.2.1. Preservation of the Endopelvic Fascia

After exploration of abdominal cavity, we first dissect the intestines adhered to the abdominal wall. This is to ensure visibility during the operation by keeping the intestines away from the pelvic cavity. After clearance of the retropubic space, the outline of the prostate is identified and the periprostatic fatty tissues are removed as much as possible. The endopelvic fascia is preserved for those with clinical stage ≤ T2c disease. However, in patients with clinical stage T3 disease and suspected periprostatic invasion, the endopelvic fascia is incised.

#### 2.2.2. No Ligation of the Deep Dorsal Vein

The deep dorsal vein complex (DVC) is not sutured. Instead, it is incised with cold scissors prior to the dissection at the prostatic apex and urethra. Incision of the DVC leads to moderate bleeding; however, the bleeding typically stops after removal of the prostate.

#### 2.2.3. Athermal Dissection of the Vas Deferens and Seminal Vesicles

The vas deferens and seminal vesicles are dissected athermally using clips. The posterior layer of the Denonvilliers' fascia is incised in an inverse U shape in the proximity of the prostate gland to the prostatic apex area.

#### 2.2.4. Complete Intrafascial Nerve Saving Technique

The main purpose of approach to prostatic dissection is cancer control and functional recovery. Therefore, the dissection plane in patients with clinical stage ≤ T2c disease is intrafascial nerve saving technique, if indicated. We develop this plane athermally by sharp and blunt dissection without the use of Hem–o–lok clips. However, 4 mm hemoclips are used if there are perforating small arteries entering the prostate capsule.

#### 2.2.5. Prostatic Apex and Urethral Dissection

**Figure 1:**
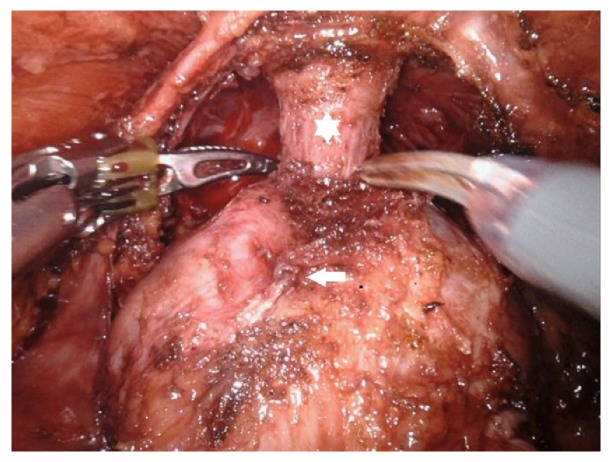
Prostatic apex and urethral dissection (urethra: white star, prostate: white arrow).

The main purpose of the prostatic apex and urethral dissection is to retain the maximum length of the urethra and to preserve the puboprostatic ligament, provided the margins are not pathologic positive. The dissection is started once the prostate is adequately mobilized. After lifting the mobilized prostate upward, the DVC is incised directly with cold scissors, which exposes the urethra. We identified the distinct plane between the prostate apex and the urethra by sweeping the apex away from the urethra (Figure 1).

#### 2.2.6. Pelvic Lymph Node Dissection

**Figure 2:**
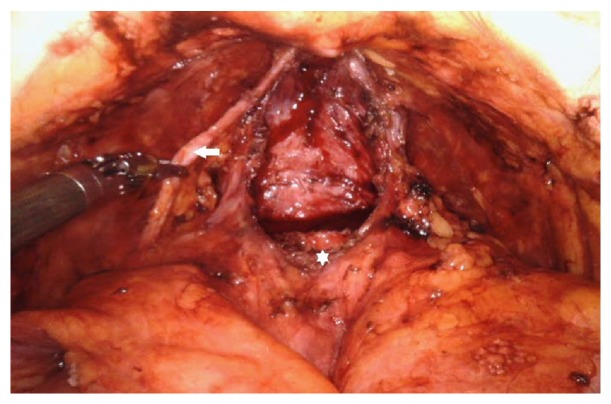
After prostate whole dissection (accessory artery: white arrow, bladder neck opening: white star).

After prostate whole dissection (Figure 2), we performed bilateral standard pelvic lymph node dissection, if indicated: prostate-specific antigen (PSA) ≥ 10 ng/mL or Gleason score ≥ 7 or clinical stage ≥ T3. Hem-o-lok clips are used during lymph node dissection instead of cauterization to prevent lymphocele formation.

#### 2.2.7. Bladder Reconstruction and Detrusorrhaphy Technique

**Figure 3:**
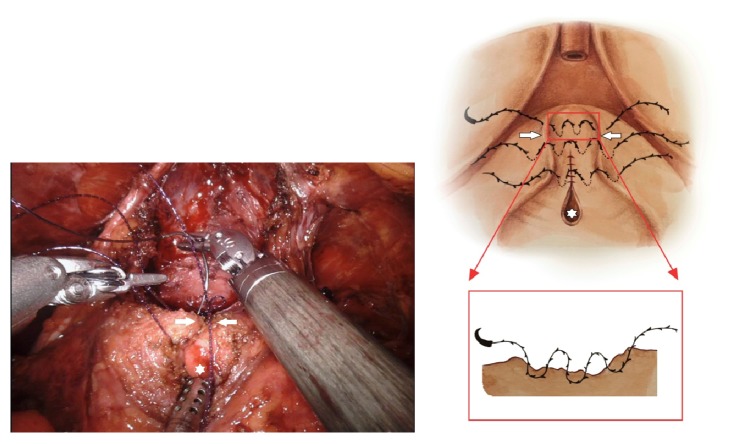
Operative and schematic view of the detrusorrhaphy technique by a flap of dynamic detrusor cuff muscles (detrusor muscles: white arrow, bladder neck opening: white star).

**Figure 4:**
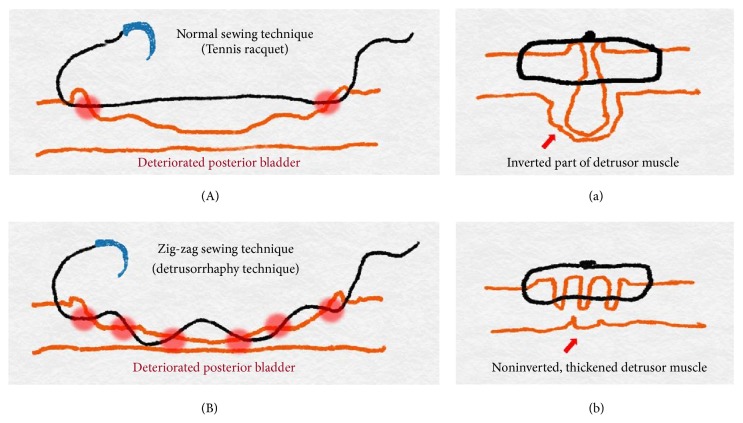
Schematic illustrations of the detrusorrhaphy technique. (A) In the standard narrowing technique, which simply sutures both wings of the dissected bladder and (a) some of the sutured detrusor muscles are inverted and do not contribute to continence recovery and may be discarded. (B) The point of the detrusorrhaphy technique is “zigzag” suturing and (b) this aims to reconstruct a physiologically and anatomically ideal form to thicken and strengthen the deteriorated muscles of detrusor during the posterior dissection of the bladder.

The anterior dissected bladder is held and pulled back by the fourth robotic arm to identify the bladder opening and posterior part of the dissected bladder. We designed the detrusorrhaphy technique based on the hypothesis that detrusor muscles would be functionally reinforced by anatomically correct reconstructions. First, the widened posterior part of the bladder opening is closed with a tennis racquet stitch using a 3–0 V–Loc suture enough to accommodate an 18Fr Foley catheter in the opposite direction after checking the trigone area inside the widened bladder opening and the ureteral orifices below it [12]. Second, the posterior gap behind the newly constructed bladder neck is widely covered by detrusorrhaphy using a flap of detrusor muscles from the posterior bladder neck to the bilateral dissected pedicles and approximated in the midline by a 3–0 V–Loc suture (Figure 3), which completes the detrusorrhaphy technique with support to the bladder neck [13]. The point of the detrusorrhaphy technique is “zigzag” suturing, which thickens and strengthens the deteriorated muscles of detrusor during posterior dissection of the bladder (Figure 4). It aims to reconstruct a physiologically and anatomically ideal shape of detrusor muscles. In the standard narrowing techniques, which simply suture both wings of the dissected bladder, this posterior reinforcement is based on the principles of Parsons and colleagues [14].

### 2.3. Data Collection

Demographic data and preoperative and postoperative functional and oncological results were compared between the two groups. Complications were recorded and evaluated using the Clavien-Dindo classification [15]. Recurrent cancer was defined according to the American Urological Association guidelines as two consecutive PSA values > 0.2 ng/mL and rising [16].

The preoperative functional parameters were assessed by the International Prostate Symptom Score (IPSS) score with Urinary Incontinence Quality of Life Scale questionnaires.

The postoperative continence was evaluated using the Expanded Prostate Cancer Index Composite survey question [17]. A patient was defined as continent if he answered “0 pad” per day. In all patients, catheter was removed at postoperative 7 days. Urinary outcomes were assessed by measuring the number of pads for 24 h and the weight of pads in patients with urinary incontinence at 0 day, 1 week, 4 weeks, 8–12 weeks, and 6 months after catheter removal. We also evaluated the IPSS score and performed uroflowmetry. Finally, the relationships between the surgeon's learning curve and the recovery of continence were analyzed by comparing the subgroups in group 1 (enrolled numbers 1–25, 26–50) and group 2 (enrolled numbers 1–23, 24–45) according to a time criterion.

The characteristics of patients were analyzed using Student's t-test or the Mann-Whitney rank sum test. Proportions were compared using chi-square test. Continuous variables were reported as the median values and interquartile range (IQR). The frequencies and proportions of categorical variables were reported as percentages. A p value of < 0.05 was considered indicative of statistically significant differences. SPSS 22.0 for windows (IBM® SPSS® version 22.0, IBM, Armonk, NY, USA) was used for all statistical analyses.

## 3. Results

### 3.1. Demographics

This study included 95 patients. Their baseline demographic and clinical data are summarized in Table 1. No significant between-group differences were observed regarding preoperative demographic and clinical data.

### 3.2. Operative Outcomes and Complications

Mean operative times in groups 1 and 2 were 250 and 220 min, respectively. The median operating time was comparable in the two groups. The estimated blood loss, blood transfusion rates (groups 1 and 2: 4 and 6.7%, respectively), mean number of days with urinary catheter, and overall complication rates were similar between the groups (Table 2).

Intraoperative complications were not encountered in any patient. During postoperative 12-month period, none of the patients had urinary retention. Moreover, there were no complications such as hematoma or lymphocele that required further procedures.

### 3.3. Continence Outcomes

**Figure 5:**
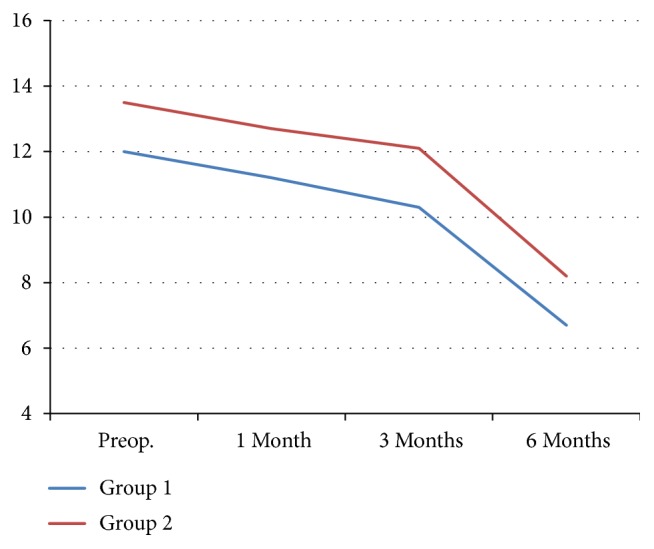
Comparison of the IPPS score between group 1 (detrusorrhaphy) and group 2 (no detrusorrhaphy) at preoperative, 1-, 3-, and 6-month follow-ups.

Continence rates in groups 1 and 2 were 68% and 0% at 0 day, 78% and 17.8% at 1 week, 86% and 64.4% at 4 weeks, 92% and 73.3% at 8–12 weeks, and 100% and 91.1% at 6 months follow-up after catheter removal, respectively (Table 3). Up to 12 weeks, the continence recovery rate in group 1 was significantly higher than that in group 2 (p < 0.05). Regarding the learning curve analysis, a progressive change in the number of continent patients and operative time in groups 1 and 2 at each time point was not recorded. We also evaluated continence using the IPSS score. There were no significant between-group differences regarding preoperative IPSS scores (12 and 13.5, respectively). At the 1-, 3-, and 6-month postoperative follow-ups, the IPSS scores were comparable in the two groups (11.2 and 12.7; 10.3 and 12.1; and 6.7 and 8.2, respectively; p > 0.1) (Figure 5).

Univariate analysis revealed a statistically significant difference in the recovery of continence at the time of catheter removal in relation to the complete nerve sparing technique difference (p = 0.036). The D'Amico risk classification appeared to influence the continence recovery at 1 week, 4 weeks, and 8–12 weeks (p < 0.05).

Multivariate analysis in group 1 showed that the patients in the D'Amico low risk and a bilateral complete nerve sparing technique group had a statistically significant advantage in terms of continence recovery at the time of catheter removal (p = 0.025). At 1 week and 4 weeks, a prostate volume < 60cc and the D'Amico low risk group indicated patients with continence recovery (p = 0.042 and p = 0.012, respectively). However, at 12 weeks, the only independent predictor variable was a low or intermediate D'Amico risk group (p = 0.028).

### 3.4. Pathologic Findings

Histopathologic data are presented in Table 2. The two groups had no significant differences in their pathologic stage, frequency of positive surgical margin (groups 1 and 2: 26% and 22.2%, respectively; p > 0.05), and Gleason score of the surgical specimen. However, the positive margin rate in the cohort of pT2-staged patients decreased to 10% and 11.1% in groups 1 and 2, respectively.

## 4. Discussion

Robotic prostate surgery in the pelvic cavity confers several advantages in terms of technical operative procedures and postoperative functional results. Thus, the oncological and functional outcomes of robotic prostatectomy may be superior to those achieved with traditional surgical methods [18]. Nevertheless, erectile dysfunction and postprostatectomy incontinence are common adverse effects of robotic prostatectomy. Postoperative incontinence is a particularly common complication that significantly affects the QoL of patients. The numerous potential causes of incontinence after RARP are associated with the disruption of normal anatomic contributors to continence [19]. These include shortening and thinning of the membranous urethra, devascularization or partial sphincter excision, bladder hypermobility and pelvic floor descent, posterior support disruption, and nerve injury. The physiological mechanisms related to postprostatectomy urinary continence are still not completely understood.

Several surgical adaptations to improve the QoL of patients have been described. Various techniques, such as the “Rocco stitch,” nerve sparing technique, preservation of the bladder neck and maximum length of the urethra, preserving the puboprostatic ligament and endopelvic fascia, anterior reconstruction, posterior rhabdosphincter reconstruction, and total anatomical reconstruction for incontinence, have been introduced as long as oncological outcomes are not compromised [8–11]. The mechanism of continence recovery after surgery is complex and not completely understood. However, it is universally accepted that maximal preservation of the original anatomic structures associated with the prostate is the key to ensure continence recovery.

At our medical center, we have employed several techniques to reduce postoperative incontinence over the last 10 years. However, we did not achieve satisfactory results. Therefore, we identified key surgical steps and established standard perioperative protocols during RARP.

We particularly focused on the detrusorrhaphy technique. This procedure is specially designed for thickening and strengthening of detrusor muscles from the posterior bladder neck to the bilateral dissected pedicles area. The “zigzag” suturing of the detrusorrhaphy technique has a morphologically fundamental difference from the traditional tennis racquet procedure, which has been simply used to reconstruct the wide-opened bladder neck. Our hypothesis aims to reconstruct a physiologically and anatomically ideal form of the detrusor muscles. In the standard narrowing techniques, which simply suture both wings of the dissected bladder, some of the sutured detrusor muscles are inverted; these muscles do not contribute to continence recovery and may be discarded. Previous reconstructions are not anatomically perfect.

First, the zigzag suture is characterized by setting the suture direction according to the contraction direction of the detrusor muscle, thereby increasing the thickness and strength of the muscle and seeking to rebuild the bladder so as not to distort its original shape. Second, the zigzag suture has been proven to be a safe and feasible procedure in that no single ischemic necrosis has occurred. Third, we searched previous literature regarding detrusor cuff reinforcement. Normally, the puboperinealis muscle forms a dynamic cuff that pinches and angulates the urethra. This cuff is often disrupted during posterior apical dissection, which weakens this support. The fourth novel aspect of the detrusorrhaphy technique involves dynamic detrusor cuff detrusorrhaphy, which supports the proximal urethra and bladder neck with contractile detrusor tissue and constricts this outlet [20]. Reconstruction of this detrusorrhaphy technique is thought to reduce stress urinary incontinence by preventing hypermobilization of the bladder neck area and is believed to be very important for continence recovery.

Our technique aimed to achieve an even earlier return to no pad state continence (≤ 12 weeks) with the potential for immediate continence. The detrusorrhaphy technique is founded on a simple assumption. The reinforcement of the posterior bladder neck correlates with functional normalcy. We attempted to reconstruct the pelvic anatomy after RARP with the goal of improving the postoperative functional status and specifically to achieve early continence.

Our results showed very early continence rates of 68%, 78%, 86%, 92%, and 100% at 0 day, 1 week, 4 weeks, 8–12 weeks, and 6 months follow-up after catheter removal, respectively. Our results are consistent with those of other studies that showed the benefits of early urinary incontinence recovery, although surgical techniques and continence definitions vary. In a nonrandomized single-arm study, Porpiglia et al. [11] achieved similar continence rates (71.8%, 77.8%, 89.3%, 94.4%, and 98.0% at 24 h and 1, 4, 12, and 24 weeks, respectively, after catheter removal).

Our study aimed to determine whether the detrusorrhaphy technique with additional surgical techniques including the use of a meticulous urethral approach for retaining the maximum length of the urethra, preservation of the puboprostatic ligament, and other factors such as nerve sparing and D'Amico risk groups could further improve the continence rate. As seen, in the multivariate analysis, the nerve sparing technique, D'Amico risk groups, and prostate volume were involved in the early recovery of urinary continence because they reasonably affected the preservation of anatomical structures and the involvement of the sacral plexus.

None of our patients developed urinary leakage or stenosis at the site of anastomosis. These findings suggest that meticulous urethrovesical anastomosis and posterior bladder neck reconstruction ensure a watertight anastomosis without a concomitant increase in the risk of stricture. Moreover, the new technique did not seem to affect the oncological results. While the overall positive surgical margin rate (groups 1 and 2: 26% and 22.2%, respectively) was higher in the previously reported study [21]. However, the positive margin rate in the cohort of pT2-staged patients (groups 1 and 2: 10% and 11.1%, respectively) was similar to or lower than the other reported study [22].

Some limitations of the present study should be acknowledged. These include the small sample size, the single-institution scope of the study, and the fact that only one surgeon was performing RARP. The continence outcomes of our study may be influenced by the extensive experience of the surgeon. Preexisting comorbidities, such as diabetes mellitus and smoking history, which could potentially affect the continence status, were not recorded. Therefore, we should perform multivariate analysis using the various factors described above in future studies.

## 5. Conclusions

Our study showed significantly better outcomes than those achieved with the standard technique in terms of urinary incontinence. The use of the detrusorrhaphy technique during RARP is simple, safe, and feasible. Although our findings need to be validated further, the technique described is relatively simple and reproducible and may be applicable for RARP.

## Figures and Tables

**Table 1:** Preoperative data in group 1 (detrusorrhaphy) and group 2 (no detrusorrhaphy).

	Group 1	Group 2	p value
Patients, number	50	45	
Age, median (IQR), year	63.5 (53.0-78.0)	65.5 (58.0-79.0)	0.684
BMI, median (IQR), kg/m^2^	25.4 (24.4-27.7)	25.8 (23.8-28.0)	0.957
ASA score, median (IQR)	2.0 (1.0-2.0)	2.0 (1.0-2.0)	0.873
TRUS, median (IQR), cc	38.6 (21.0-105)	36.5 (22.5-70.5)	0.425
PSA, median (IQR), ng/ml	8.9 (3.2-42.0)	9.5 (4.3-52.5)	0.070
Biopsy Gleason score, median (IQR)	7 (6-9)	7 (6-9)	0.895
IPSS score, median (IQR)	12 (3.0-21.0)	13.5 (2.0-20.0)	0.472
IIEF-5 score, median (IQR)	18.0 (13.5-22)	18.5 (12.5-21.0)	0.775
D'Amico risk group (%)			
(i) Low risk	35 (70)	28 (62.2)	0.065
(ii) Intermediate risk	10 (20)	9 (20)	0.855
(iii) High risk	5 (10)	8 (17.8)	0.073

IQR: interquartile range; BMI: body mass index; ASA score: American Society of Anesthesiologist score; TRUS: transrectal ultrasound; PSA: prostate-specific antigen; IPSS: International Prostate Symptoms Score; IIEF: International Index of Erectile Function.

**Table 2:** Perioperative and histopathologic data in group 1 (detrusorrhaphy) and group 2 (no detrusorrhaphy).

	Group 1 (n=50)	Group 2 (n=45)	p value
Operative time, median (IQR), min	250 (180-300)	220 (150-300)	0.275
Blood loss, median (IQR), ml	200 (80-400)	200 (100-600)	0.895
Nerve sparing (%)			
(i) Bilateral	30 (60)	24 (53.3)	0.085
(ii) Unilateral	12 (24)	12 (26.7)	
(iii) None	8 (16)	9 (20)	
PLND (%)	40 (80)	38(84.4)	0.125
Complications (%)			
(i) Clavien grade 1			
(ii) Clavien grade 2	2 (4)	3 (6.7)	0.089
(iii) Clavien grade 3	0	0	
Pathologic stage (%)			
(i) pT2	35 (70)	23 (51.1)	0.084
(ii) pT3a	10 (20)	12 (26.7)	0.126
(iii) pT3b	5 (10)	10 (22.2)	0.074
Pathologic Gleason score (%)			
(i) <6	10 (20)	9 (20)	
(ii) 7	30 (60)	24 (53.3)	0.245
(iii) >8	10 (20)	12 (26.7)	0.125
Positive surgical margins (%)	13 (26)	10 (22.2)	0.095
(i) pT2	5 (10)	5 (11.1)	0.185
(ii) pT3	8 (16)	5 (11.1)	0.07
Positive PLND (%)	0	0	

IQR: interquartile range; PLND: pelvic lymph node dissection.

**Table 3:** Continence data at various follow-up points in group 1 (detrusorrhaphy) and group 2 (no detrusorrhaphy).

Time	Patients achieving continence, n (%)		p value
	Group 1 (n=50)	Group 2 (n=45)	
0 day	34 (68.0%)	0 (0%)	< 0.001*∗*
1 week	39 (78.0%)	8 (17.8%)	< 0.001*∗*
4 weeks	43 (86.0%)	29(64.4%)	< 0.001*∗*
8-12 weeks	46 (92.0%)	33 (73.3%)	0.043*∗*
6 months	50 (100%)	41 (91.1%)	0.089
12 months	50 (100%)	43 (95.6%)	0.115

*∗* significant at p < 0.05

## Data Availability

The data used to support the findings of this study are available from the corresponding author upon request.

## Abbreviations and Acronyms

RARP: Robot-assisted radical prostatectomy
QoL: Quality of life
DVC: Dorsal vein complex
PSA: Prostate-specific antigen
IPSS: International Prostate Symptom Score
IQR: Interquartile range.
